# Experimental study on rubber concrete filled steel tube members under pure bending

**DOI:** 10.1038/s41598-022-13659-3

**Published:** 2022-06-07

**Authors:** Hongshuang Wu, Cong Wang, Yikui Bai, Shiyu Tong, Yanhua Liu

**Affiliations:** College of Water Conservancy, Shenyang Agricultural University, Shenyang, 110866 People’s Republic of China

**Keywords:** Civil engineering, Mechanical engineering

## Abstract

The test of four rubber concrete filled steel tube (RuCFST) members, one concrete filled steel tube (CFST) member and one empty member were conducted under pure bending. The main parameters were the shear span ratio (*λ*) from 3 to 5, and the rubber replacing ratio (*r*) from 10% to 20%. The bending moment-strain curves, the bending moment-deflection curves and the bending moment–curvature curves were obtained. The failure modes of core rubber concrete were analyzed. The failure mode of RuCFST members was bending failure from the results. The cracks of rubber concrete were distributed evenly and sparsely, and the filling of rubber in core concrete prevented the development of cracks. The shear span ratio has little effect on the behavior of the tested specimens. While the rubber replacing ratio had little effect on the bending moment capacity, but had some influence on the bending stiffness of the tested specimens. After filling in rubber concrete, the bending moment capacity and the bending stiffness can be improved compared with the empty steel tube specimen.

## Introduction

Due to the good seismic performance and high bearing capacity, traditional concrete filled steel tube (CFST) structures are widely used in modern engineering practices^1–3^. As a new type of concrete-rubber concrete, rubber particles are used to partially replace natural aggregates. The rubber concrete filled steel tube (RuCFST) structures are formed by filling rubber concrete into steel tubes, which improve ductility and energy efficiency of the composite structures^4^. It is not only a way to play the excellent performance of CFST members, but also effectively utilize waste rubber, which meets the development needs of green circular economy^5,6^.

For the past few years, scholars have studied deeply on the behaviors of traditional CFST members under axial load^7,8^, axial load-moment interaction^9–11^ and pure bending^12–14^. The results show that the flexural capacity, stiffness, ductility, and energy dissipation ability are all strengthened and showed a good ductility failure of the CFST columns and beams due to the filling concrete of the interior.

At present, the behavior and performance of RuCFST columns under combined axial load have been studied by some researchers. Liu and Liang^15^ conducted some experiments on RuCFST stub columns, and the bearing capacity and stiffness decreased with increasing of the rubber substitution ratio and the rubber particle size, while ductility increased compared with CFST columns. Duarte^4,16^ tested some short RuCFST columns, and the results showed that RuCFST columns had higher ductility with the increase of the rubber aggregates content. Similar results were also reported by Liang^17^ and Gao^18^ on the properties of ordinary and thin-walled RuCFST stub columns. Gu et al.^19^ and Jiang et al.^20^ studied the bearing capacity of RuCFST members at high temperature. The results indicated that the addition of rubber strengthened the ductility of structures. As the temperature rises, the bearing capacity decreases slightly at first. Patel^21^ analyzed the compression and bending behavior of short round-ended concrete filled steel tubular beam-columns under axial and uniaxial loads. The computational simulation and parametric analysis indicated that the fiber-based modeling strategies can accurately investigate the performance of short RCFST. The bending capacity increases with the increase of aspect ratio, steel and concrete strengths, decreases with the increase of depth-to-thickness ratio. Generally speaking, the RuCFST stub columns have similar behavior to those of CFST columns, and better ductility than that of CFST columns.

From the above review, it can be found that RuCFST columns became better when rubber additive was used properly in the core concrete of CFST columns. Since there is no axial load, pure bending is an extremity of column-beam. In fact, the bending performance of RuCFST was not independent of the performance of axial load^22^. In practical engineering, RuCFST structure is often beard bending load. The study of its pure bending performance is helpful to determine the deformation and failure mode of RuCFST members under earthquake^23^. For RuCFST structures, it is necessary to study the pure bending properties of RuCFST members.

In view of this, the test of six specimens was tested to explore the mechanical properties of square RuCFST members subject to pure bending. The rest of the paper is arranged as follows. Firstly, six specimens with square sections filled with or without rubber concrete were tested. The failure modes of each specimen were observed, and the test results will be obtained. Secondly, the properties of RuCFST members subjected to pure bending were analyzed, as well as the influences of shear span ratio from 3 to 5, and the rubber replacing ratio from 10 to 20% on the performances of RuCFST structures were explored. Finally, the differences of capacities and flexural stiffness between the RuCFST members and traditional CFST members were compared.

## Experimental setup

Six CFST specimens were conducted, four of them were filled with rubberized concrete and one was filled with conventual concrete while the sixth one was left empty. The effect of rubber replacing ratio (*r*) and shear span ratio (*λ*) were discussed. The main parameters of specimens are given in Table 1. The letter *t* represents the thickness of the tube; *B* is the side length of the specimens; *L* is the height of the specimens; *M*_ue_ is the measured bending bearing capacity; *K*_ie_ is the initial bending stiffness; *K*_se_ is the bending stiffness in the service phase.

**Table 1:** Parameters of specimens.

Number	*B* × *t* × *L* (mm)	*λ*	*r* (%)	*M*_ue_ (kN·m)	*K*_ie_ (kN m^2^)	*K*_se_ (kN m^2^)
SB1	140 × 3 × 2000	5	20	27.35	904.14	903.96
SB 2	140 × 3 × 2000	4	20	25.55	894.74	873.23
SB 3	140 × 3 × 2000	3	20	26.51	840.18	814.52
SB 4	140 × 3 × 2000	5	10	28.66	1314.19	1057.32
SB 5	140 × 3 × 2000	5	0	27.78	1104.09	977.54
SB 6	140 × 3 × 2000	5	-	16.19	773.43	643.42

The RuCFST specimens were made of four steel plates which were welded in pairs to form a hollow square steel tube, then filled with concrete. At each end of the specimen, one steel plate with 10 mm thick was welded. The mechanical performances of steel were listed in Table 2. According to the requirements of China Standards GB/T228-2010^24^, the ultimate strength (*f*_*u*_ ) and yield strength (*f*_*y*_) of the steel tube were measured by standard tensile coupon test. The test results were 260 MPa and 350 MPa, respectively. The elastic modulus (*E*_s_) was 176GPa, and the Poisson-ratio (*ν*) of the steel was 0.3.

**Table 2:** Mechanical performance of steel.

*f*_y_ (MPa)	*f*_u_ (MPa)	*E*_s_ (GPa)	*ν*
260	350	176	0.3

In the test, the reference concrete compressive cube strength (*f*_cu_) at the 28 days was designed as 40 MPa. Based on the previous reference^25^, the shear span ratios of 3, 4 and 5 were selected because this may reveal any problems of shear transfer. Two types of rubber replacement ratio 10% and 20% to substitute the sand of the concrete mixture. Ordinary tire rubber powder from cement factory of Tianyu (brand Tianyu, China) was used in this study. The rubber particle size was 1-2 mm.The rubberized concrete and mixture proportions are given in Table3. For each type of mixed rubberized concrete, three 150 mm cubes were poured and cured under the test conditions required by the specification. The sand used in the mixture was siliceous sand, and the coarse aggregate was carbonatite from Shenyang City, Northeast China. The 28 day’s compressive cube strength (*f*_*cu*_), prismatic compressive strength (*f*_*c*_^’^) and elasticity modulus (*E*_c_) of different rubber replacing ratio (10% and 20%) were listed in Table 3. The properties were measured in accordance with the China Standards GB50081-2019^26^.

**Table 3:** Mixture of concrete (kg/m^3^) and material properties of rubber concrete.

Rubber replacing ratio (r)	Water	Fly ash	Cement	Sand	Aggregate	Rubber	*f*_cu_(MPa)	*f*_c_^'^(MPa)	*E*_c_(GPa)
20%	164	72	340	530	1138	60	34.60	20.10	24.7
10%	164	72	340	597	1138	30	38.20	22.41	26.0
0%	164	72	340	663	1138	0	43.40	25.76	27.7

**Figure 1:**
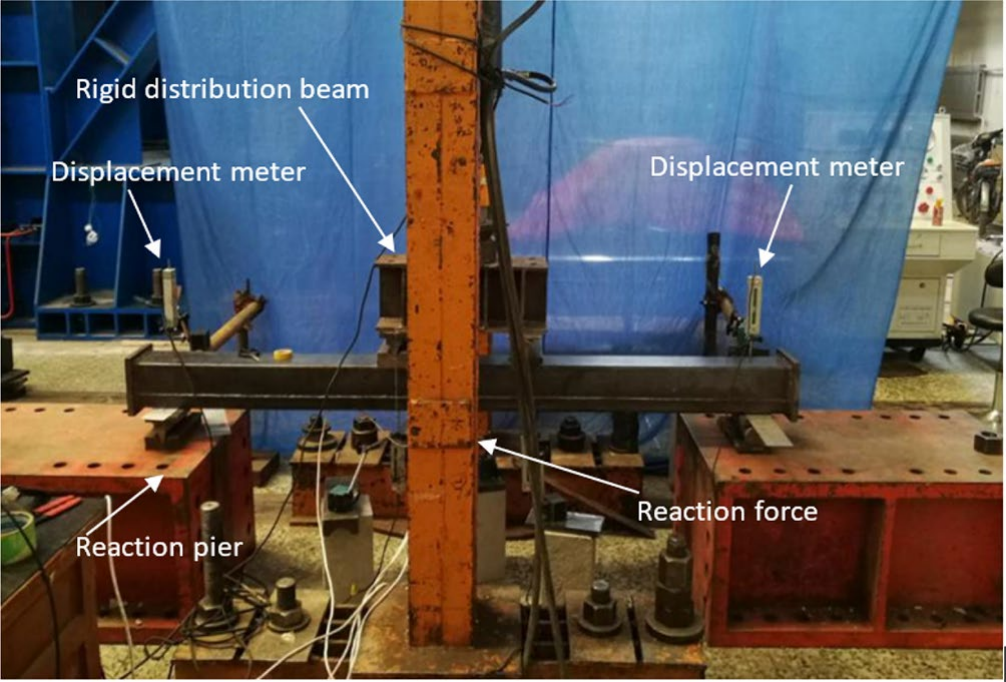
Test set-up.

**Figure 2:**
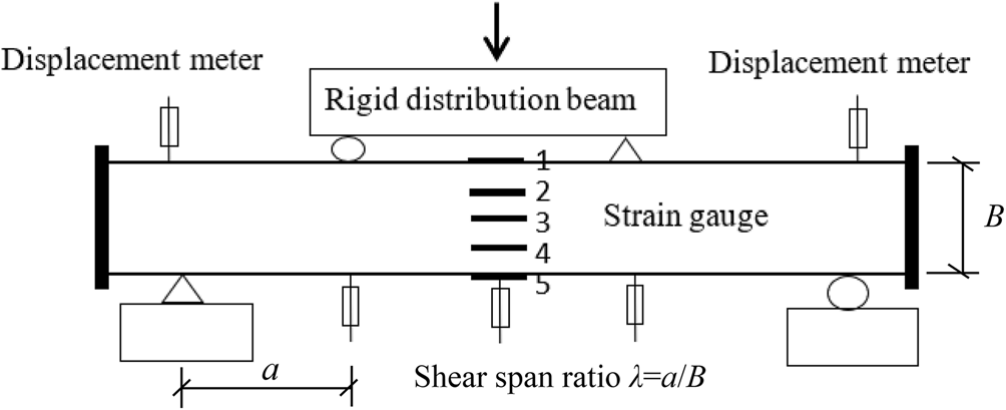
Schematic diagram of measuring device.

All specimens were tested by one 600kN hydraulic ram. During loading, two concentrated forces were applied symmetrically on the four-point bending test bed, then distributed to the specimen. The strains were measured by five strain gauges on the surfaces of each specimen. The deflection was observed by three displacement transducers shown in Figs. 1 and 2.

Preloading system was used in the test. The load was at the speed of 2 kN/s, then paused at the loading reaches 10 kN, and checked whether the instrument and strain gauge were in normal working condition. In the elastic range, each load increment was applied at less than one tenth of the predicted peak load. While the steel tube yields, the applied load at less than one fifteenth of the predicted peak load. Hold for about two minutes after each level of load was applied during the loading stage. As the specimen approached failure, the rate of continuous loading slowed down. While the axial load reached below 50% of the ultimate load or the specimen appeared obvious failure phenomena, the loading was terminated.

## Experimental results and discussion

### Failure mode

**Figure 3:**
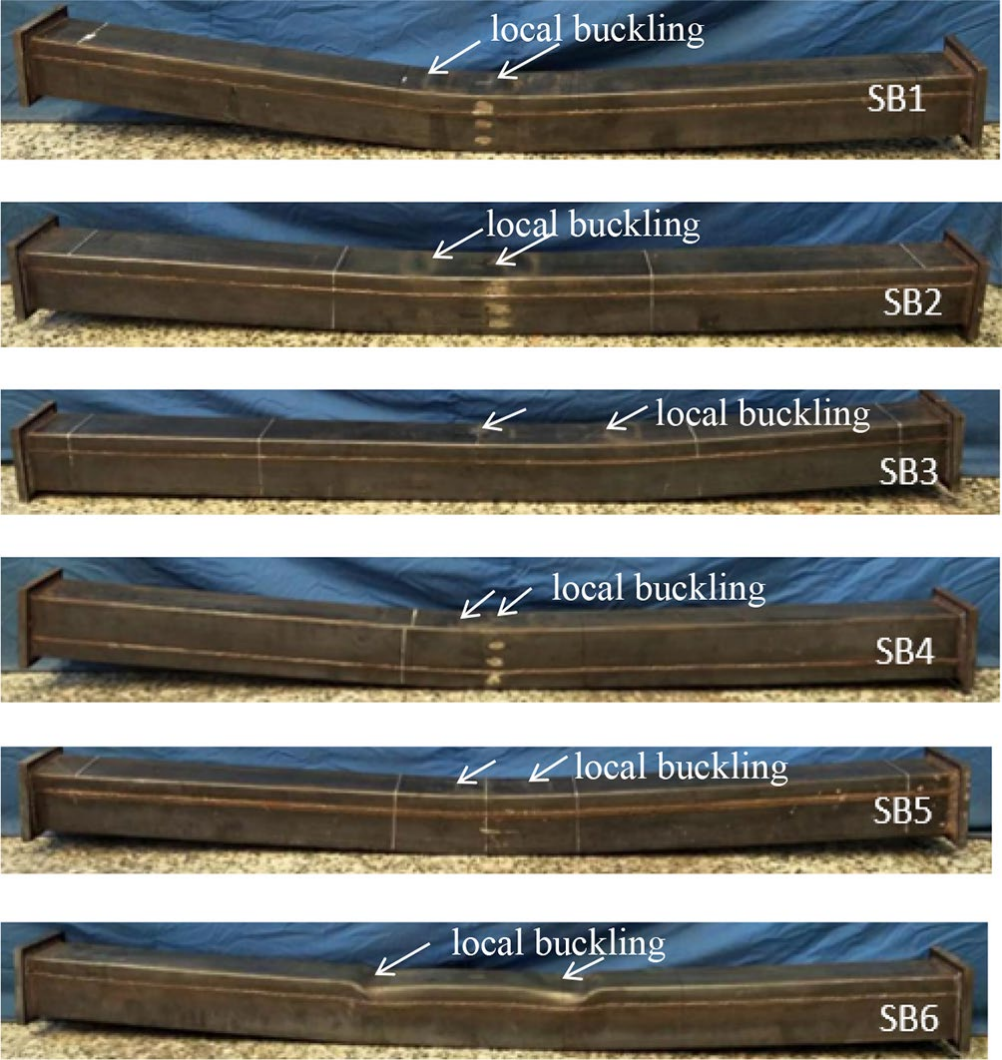
Failure modes of specimens.

The failure of all the tested specimens showed good ductility. No obvious tensile crack was observed in the tensile region on the steel tubes of specimens. The typical pattern of damage in the steel tubes were shown in Fig. 3. Taking specimen SB1 as an example, during the initial loading period, when the bending moment smaller than 18kN.m, specimen SB1 was in an elastic stage without obvious deformation, and the growth rate of measured bending moment was greater than that of curvature. Then, the steel tube in the tensile region yielded and entered the elastoplastic stage. When the bending moment reached about 26kN.m, there were some bulking began to appear in the compression region of midspan section of steel. With the increasing of load, the bulking gradually developed. The load–deflection curve did not decline until the load reached the peak points.

**Figure 4:**
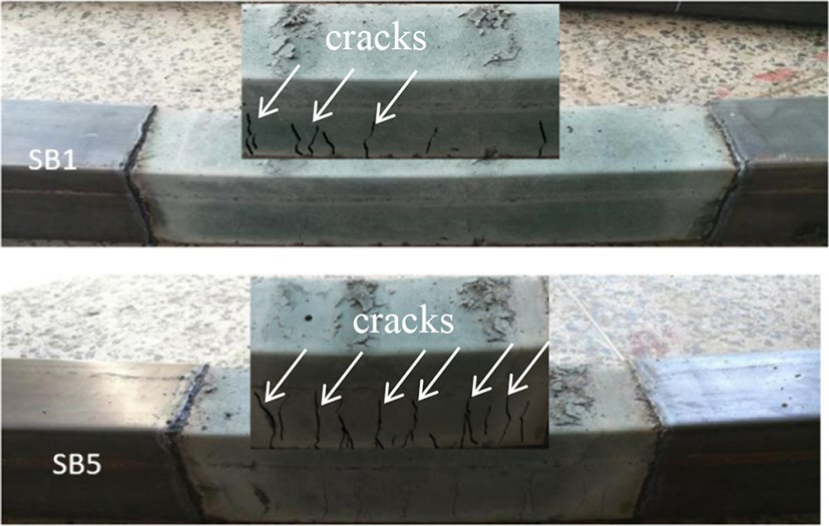
Typical failure modes of core concrete.

After the experiments completed, specimen SB1 (RuCFST) and specimen SB5(CFST) were cut open to observe the failure mode of core concrete more clearly, as showed in Fig. 4. From Fig. 4, the cracks of specimen SB1 were evenly distributed and sparsely in the core concrete, and the spacing of them were between 10 cm and 15 cm. However, the spacing of the cracks in specimen SB5 were between 5 cm and 8 cm, and the cracks were irregular and obvious. In addition, the cracks of specimen SB5 extended roughly 90° from the tensile region to the compressive zone and developed to about 3/4 of the section height. The cracks in the core concrete of specimen SB1 were smaller and sparser than that of specimen SB5. The replacement of sand with rubber can prevent concrete cracks from developing to some extent.

### Distribution of deflection curves

**Figure 5:**
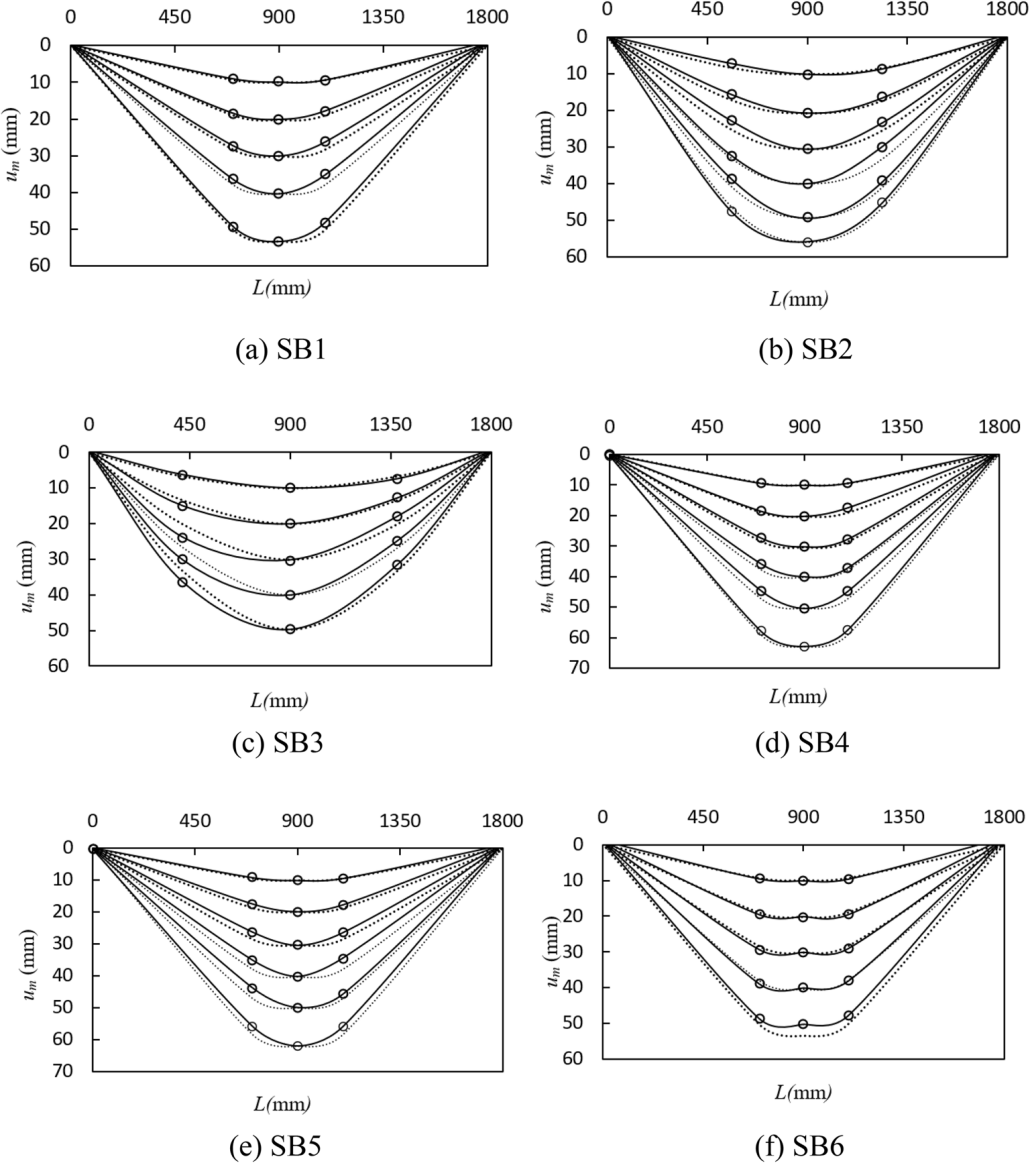
Distribution of deflection along the length of specimens.

Figure 5 shows the distribution of deflection along the length of each specimen. The solid line represents the deflection curve of the measured specimens, while the dotted line represents the sinusoidal half-wave curve. From Fig. 5, during the initial loading period, the deflection curves of components coincide well with sine half wave curves. As the load increases, the deflection curves deviate slightly from the sine half wave curves. In general, during loading, the deflection curves of all specimens at each measuring point presents a symmetrical sinusoidal half-wave curve.

### Bending moment (*M*) -curvature (*φ*) curves

Due to the deflection of RuCFST members under pure bending conforms to sine half wave curves, so the flexural equation can be represented as:

1 $$ y = u_{m} \sin \frac{\pi }{l}x $$

Take the second derivative of the above formula:

2 $$ \phi = \frac{{\pi^{2} }}{{l^{2} }}u_{m} \sin \frac{\pi x}{l} $$

In the mid-span section, $x$ is equal to *l*/2, so the formula (2) can be written out as follows:

3 $$ \phi = \frac{{\pi^{2} }}{{l^{2} }}u_{m} $$

**Figure 6:**
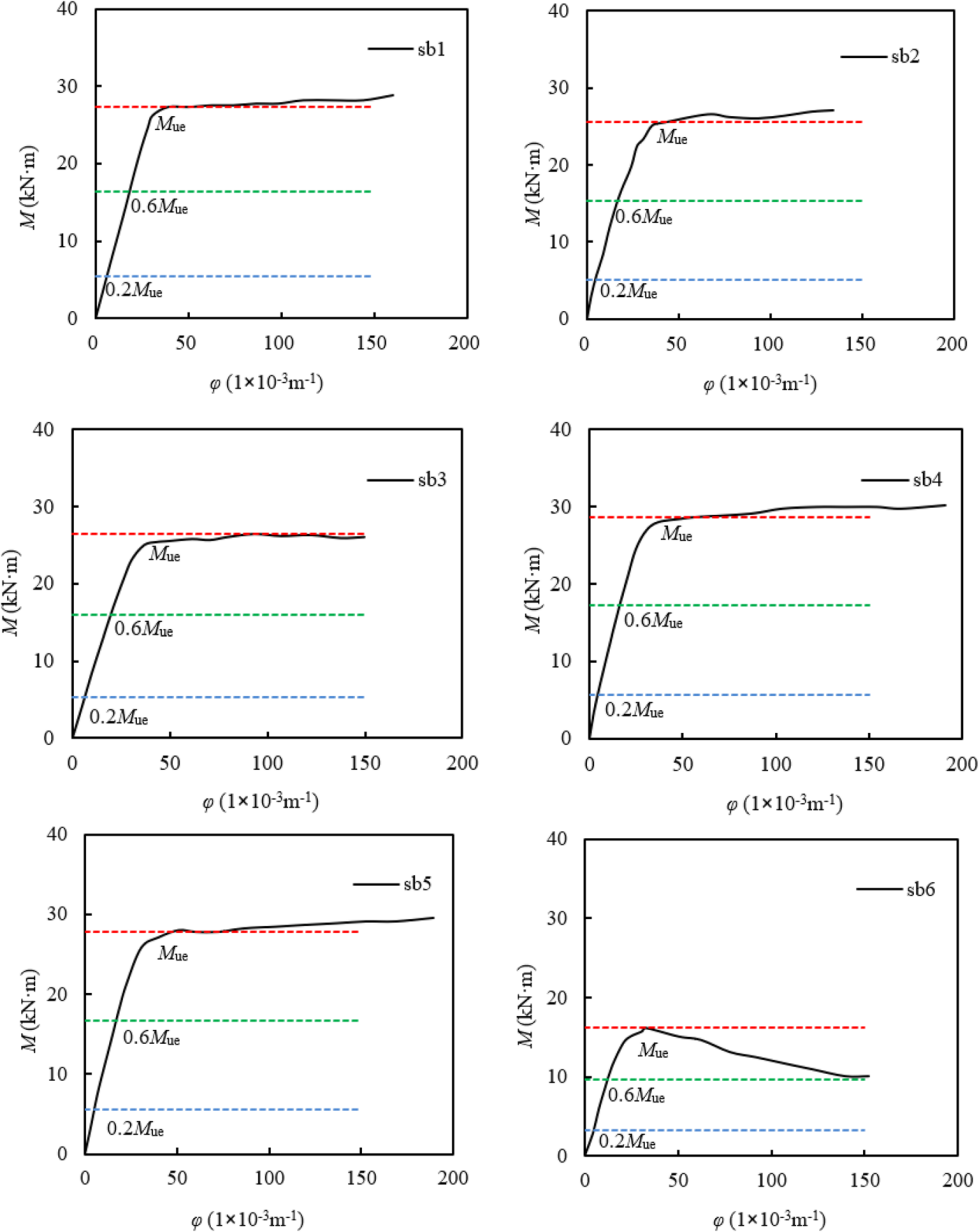
Bending moment (M) -curvature (φ) curves.

When the maximum fiber strain is 0.01, the corresponding bending moment is defined as the ultimate bending moment bearing capacity of the member, considering the practical application conditions^27^. The measured bending moment capacity (*M*_ue_) so defined in this way was given in Table 1. According to the measured bending moment capacity (*M*_ue_) and the calculation of curvature (*φ*) by formula (3), the *M*-*φ* curves can be plotted in Fig. 6. The initial stiffness *K*_ie_ is taken as the corresponding secant bending stiffness when *M* = 0.2*M*_ue_^28^. The bending stiffness (*K*_se_) in the service phase is set as the corresponding secant bending stiffness when *M* = 0.6*M*_ue_.

As seen from the bending moment–curvature curves, in the elastic stage, the bending moment and curvature showed a significant linearity increase. The growth rate of bending moment was obviously greater than that of the curvature. When the bending moment *M* was equal to 0.2*M*_ue_, the specimen reached the limit of the elastic stage. With the loading increased, the specimen had plastic deflection and entered the elastic–plastic stage. When the bending moment *M* was equal to 0.7 ~ 0.8*M*_ue_, the steel tube in tensile region and compressive region successively reached its yield. At the same time the *M*-*φ* curves of the specimen began to show an inflection point and had a nonlinear growth, and the combine effect of steel tube and core rubber concrete could be enhanced. When *M* was equal to *M*_ue_, the specimens got into the plastic strengthening stage, the deflection and curvature of the specimens increased rapidly, while the bending moment increased slowly.

### Bending moment (*M*)-strain (*ε*) curves

**Figure 7:**
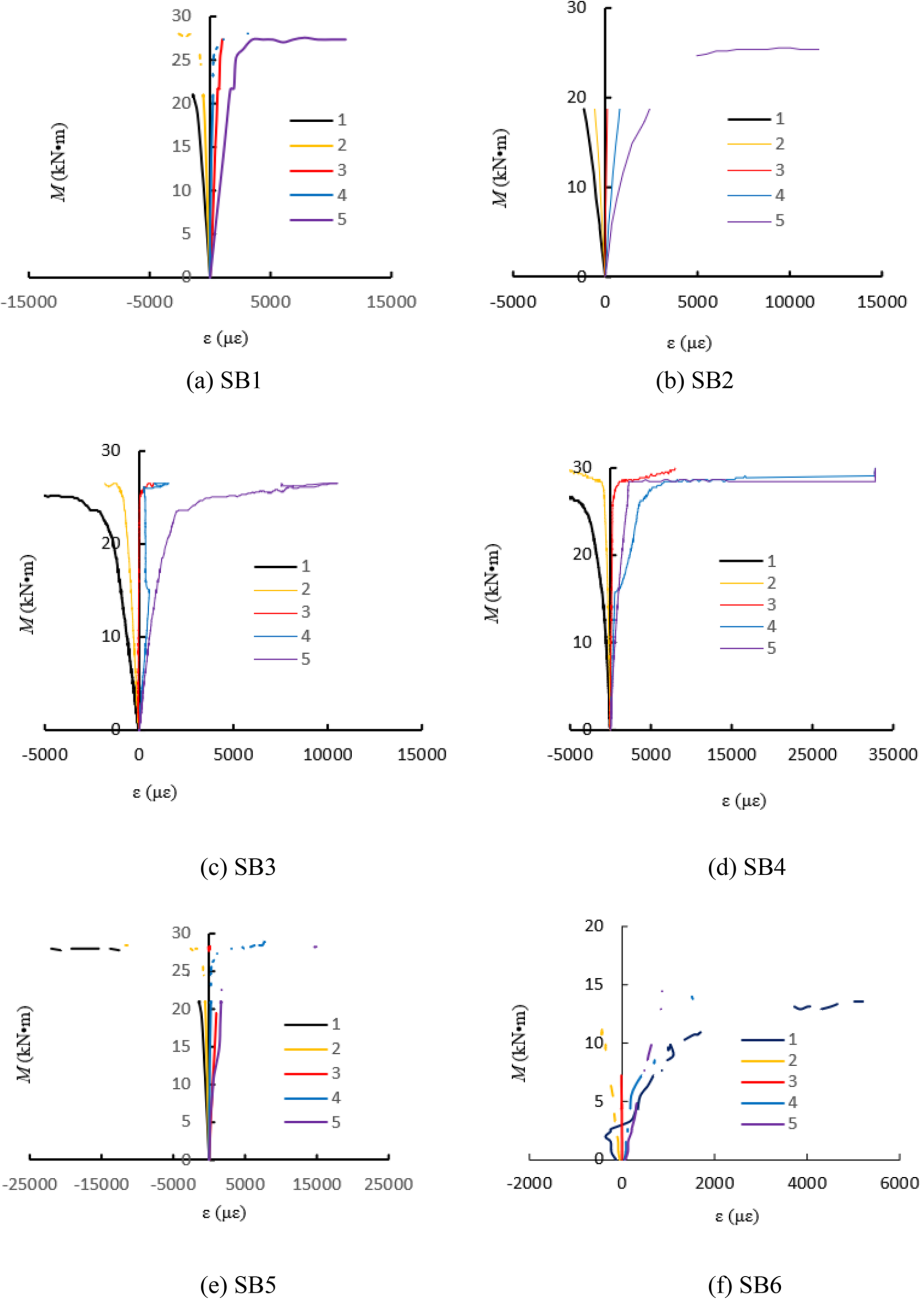
Bending moment-strain curves of specimens.

Figure 7 showed the bending moment(*M*)-strain(*ε*) curves of each specimen. The upper part of the mid-span section in the specimens was under pressure and the lower part was under tension. The strain gauges labeled “1” and “2” were located at the upper part of the specimens, and the strain gauge labeled “3” was located in the middle of the specimen while those labelled “4” and “5” were located in the lower part of the specimens as showed in Fig. 2. From Fig. 7, the longitudinal strain in the tensile and compressive zones of the members were close to each other during the initial loading period, and the strain was approximately linear. The longitudinal strain at the middle section increased slightly, but the increase was very small. Subsequently, the rubber concrete in the tensile region cracked. Because the steel tube in the tensile region needed to support the force alone, and the rubber concrete and steel tube of compressive region sustained the load together, so the strain in tensile zone of members was larger than that of the compressive zone. With the loading increased, the strain exceeded the yield strain of steel, the steel tube entered the elastic–plastic stage. The strain growth rate of specimen was obviously faster than that of bending moment, and the plastic zone began to develop to the full section.

### Bending moment(*M*)-deflection(*u*_m_) curves

**Figure 8:**
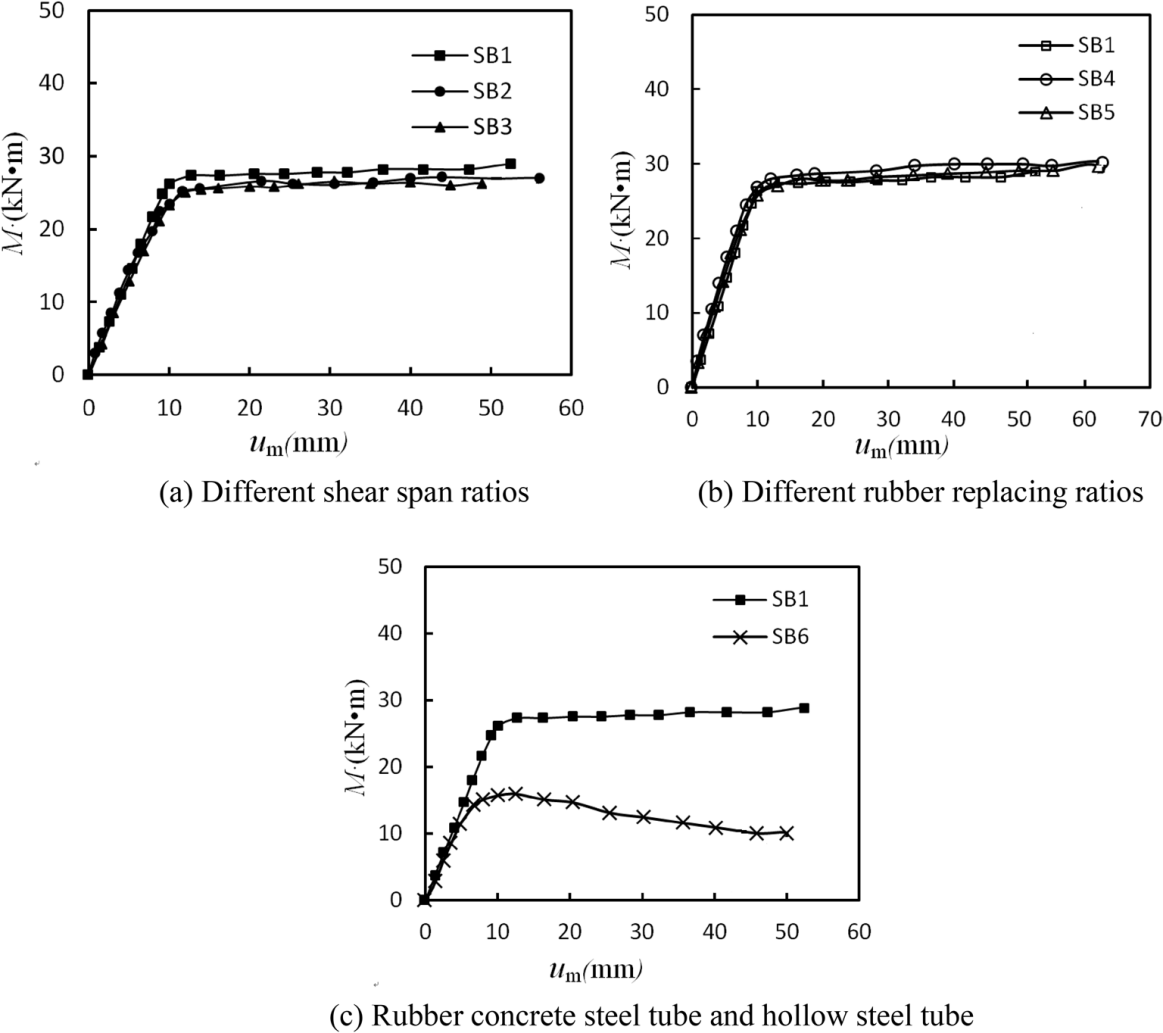
Bending moment (*M*)- deflection (*u*_m_) curves of specimens.

The *M*-*u*_m_ curves of each specimen were showed in Fig. 8. From Fig. 8, all the *M*-*u*_m_ curves followed the same trends as those of traditional CFST members^22,27^. In each case, the *M*-*u*_m_ curves showed an elastic response in the initial stage, followed by an inelastic characteristic with reducing stiffness, until the ultimate bending moment capacity was gradually reached. However, due to the different test parameters, the *M*-*u*_m_ curves had slight differences. In Fig. 8a was plotted moment-deflection for shear span ratios ranging from 3 to 5. The bending moment capacity of specimen SB2 (the shear span ratio *λ* = 4) was 6.57% lower than specimen SB1(*λ* = 5), while the bending moment capacity of specimen SB3 (*λ* = 3) was 3.76% larger than specimen SB2 (*λ* = 4). In general, the trend of the bending moment capacity was not obvious with the increasing of shear-span ratio. It seemed to the *M*-*u*_m_ curves was independent of shear span ratio. This was consistent with that have been observed by Lu and Kennedy^25^ for CFST beams with shear span ratio varying from 1.03 to 5.05. Possible causes for CFST members, the force transmission mechanism between core concrete and steel tube is almost the same under different shear span ratio, which is not as obvious as that of reinforced concrete members^25^.

From Fig. 8b, the bending moment capacity of specimens SB4 (r = 10%) and SB1(r = 20%) were slightly higher or lower than that of the traditional CFST specimen SB5(r = 0), with the increase of 3.15% and the decrease of 1.57%, respectively. While the initial bending stiffness (*K*_ie_) of specimens SB4 and SB1 were much higher than that of specimen SB5, they were 19.03% and 18.11%, respectively. The bending stiffness in the service phase (*K*_se_) of specimens SB4 and SB1 were 8.16% and 7.53% higher than that of specimen SB5 respectively. These showed that the rubber replacing ratio had little influence on the bending capacity, but greater influence on the bending stiffness of RuCFST specimens. This may be related to the fact that the ductility of rubber concrete in RuCFST specimens is higher than that of natural concrete in traditional CFST specimens. In general, the crushing and cracking of natural concrete started expanding earlier than rubber concrete^29^. From the typical failure modes of core concrete (Fig. 4), the cracks of specimen SB5 (natural concrete) were more and denser than that of specimen SB1 (rubber concrete). This may contribute to the higher confinement provided by the steel tube to specimen SB1 with rubber concrete compared to specimen SB5 with natural concrete. The study of Durate^16^ also got the similar conclusion.

From Fig. 8c, compared with hollow steel tube members, RuCFST members had better bending capacity and ductility. The bending capacity of RuCFST specimen SB1 (r = 20%) was 68.90% higher than that of empty steel tube specimen SB6, while the initial bending stiffness (*K*_ie_) and the bending stiffness in the service phase (*K*_se_) of specimen SB1 were 40.52% and 16.88% higher than that of specimen SB6. The combined effect of steel tube and core rubber concrete improved the bending capacity and stiffness of the composite members. RuCFST members exhibit good ductility specimen when subjected to pure bending load.

### Prediction of bending moment

**Figure 9:**
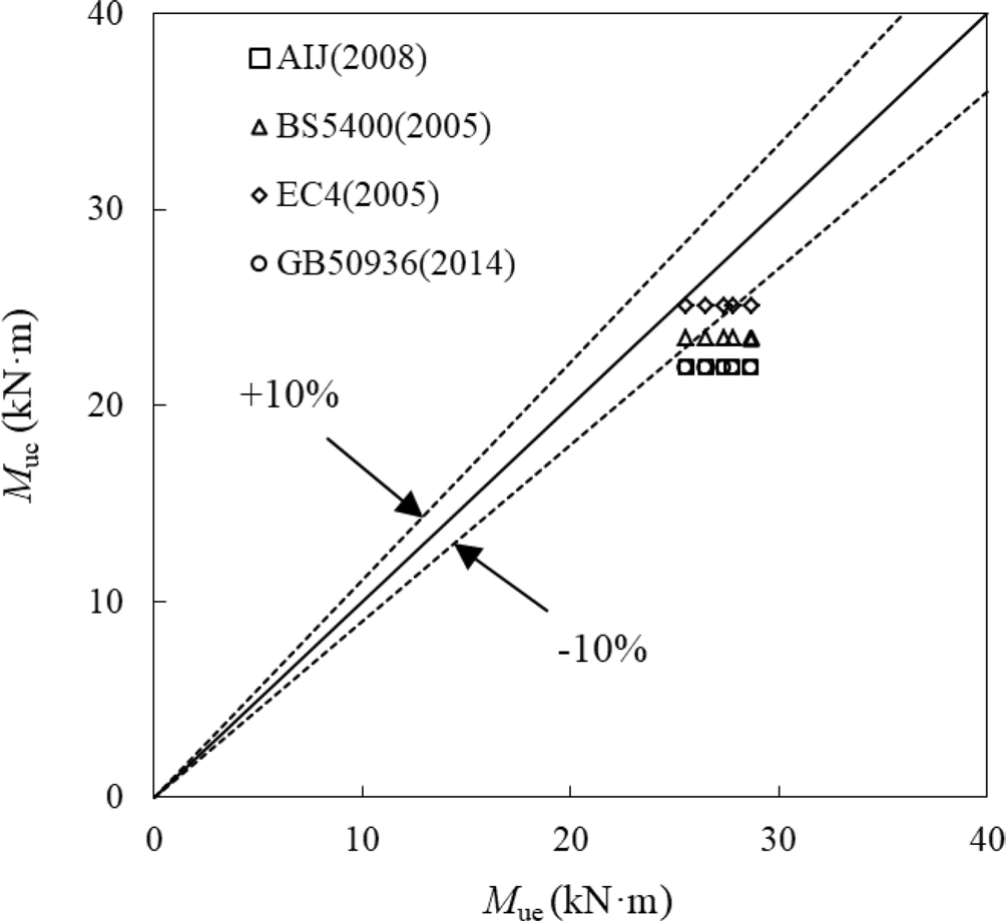
Comparison of calculated bending moment (*M*_uc_) and test bending moment (*M*_ue_).

The comparison of the obtained bending moment with those stipulated by current design standards, e.g. Japanese code AIJ(2008)^30^, British code BS5400(2005)^31^, European code EC4(2005)^32^, Chinese code GB50936(2014)^33^ were made. The ratios of the calculated bending moment (*M*_uc_) and the tested bending moment (*M*_ue_) were listed in Table 4 and plotted in Fig. 9. The calculated values of AIJ (2008), BS5400 (2005), GB50936 (2014) were 19%, 13.2% and 19.4% lower than the mean experimental values, respectively. The bending moment calculated by EC4(2005) was 7% lower than the mean test values, which was the closest.

**Table 4:** Comparison of calculated values with test values.

Number	*M*_ue_	AIJ (2008)		BS5400 (2005)		EC4 (2005)		GB50936(2014)	
		*M*_uc_	*M*_uc_/*M*_ue_	*M*_uc_	*M*_uc_/*M*_ue_	*M*_uc_	*M*_uc_/*M*_ue_	*M*_uc_	*M*_uc_/*M*_ue_
SB1	27.35	21.96	0.80	23.43	0.86	25.09	0.92	21.86	0.80
SB2	25.55	21.96	0.86	23.43	0.92	25.09	0.98	21.86	0.86
SB3	26.51	21.96	0.83	23.43	0.88	25.09	0.95	21.86	0.82
SB4	28.66	21.96	0.77	23.58	0.82	25.28	0.88	21.86	0.76
SB5	27.78	21.96	0.79	23.79	0.86	25.52	0.92	21.86	0.79
Mean		0.810		0.868		0.93		0.806	
Standard deviation		0.032		0.032		0.033		0.033

## Conclusions

The mechanical properties of RuCFST members under pure bending were experimentally studied. Within the investigation, the following conclusions can be obtained.

The tested RuCFST members had showed a similar behavior as the traditional CFST specimens. Good ductility manner has been observed for RuCFST and CFST specimens due to the infill of rubber concrete and concrete except for the empty steel tube specimen.

The shear span ratio, varying from 3 to 5, had little effect on the tested bending moment capacity and bending stiffness. The rubber replacing ratio had almost no effect on tested bending moment capacity, but had some influences on the bending stiffness of tested specimens. The initial bending stiffness of specimen SB1with a rubber replacing ratio of 10% was 19.03% higher than that of the traditional CFST specimen SB5. European code EC4(2005) can accurately estimate the ultimate bending capacity of RuCFST members. The addition of rubber in the core concrete improved the brittleness of concrete; making the RuCFST members had good ductile manners.

## Data Availability

The data used in the study are available from the corresponding author upon request.
